# Popliteal Pterygium Syndrome: A Rare Entity

**Published:** 2012-03-01

**Authors:** Muhammad Qasim, Mahmood Shaukat

**Affiliations:** Department of Paediatric Surgery, Mayo Hospital Lahore, Pakistan

**Keywords:** Popliteal pterygium syndrome, Autosomal dominant disorder, Familial

## Abstract

The popliteal pterygium syndrome is a congenital malformation that includes orofacial, musculoskeletal and genitourinary anomalies. It is a rare autosomal dominant disorder. We report one family with popliteal pterygium syndrome affecting father and his two daughters, who underwent surgical corrections for multiple congenital malformations.

## INTRODUCTION

Popliteal pterygium syndrome (PPS) is a rare autosomal dominant disorder. Its incidence is approximately 1 in 300000 live births. It was first described by Trelat in 1869. The term PPS was coined by Gorlin et al in 1968 on the basis of the most unusual anomaly, the popliteal pterygium [1-4].

The clinical features of the syndrome are highly variable and show different combinations of anomalies like cleft palate, cleft lip, lower lip pits or sinuses, popliteal webbing, syndactyly, genitourinary anomalies, nail anomalies, syngnathia, ankyloblepharon, talipes, and digital reduction defects [1-4]. The familial nature of the disease and follow up of complete family are the reasons of reporting.

## CASE REPORT

A 6 year old girl, diagnosed case of popliteal pterygium syndrome, was admitted to our unit for pharyngoplasty. She had a series of surgeries for cleft lip and palate, lower lip sinus and bilateral popliteal pterygium (Fig. 1). Bilateral labioplasty was done at the age of 3 months, release of left and right knee flexion contracture at 7 months and 10 months of age, respectively (Doppler ultrasound showed no vessels in the band). Per-operatively, no nerve was found in the fibrous band. At 15 months of age mucous membrane adhesions were released (upper and lower buccal mucosa to the tongue). Palatoplasty was performed at the age of 1 ½ year. Left leg contracture was severe and had a repeat release at the age of 2 years. Soft tissue lengthening was done by releasing the fibrous band and lengthening of skin was made by doing Z-plasty. At the same time excision of lower lip pit was done.

**Figure:**
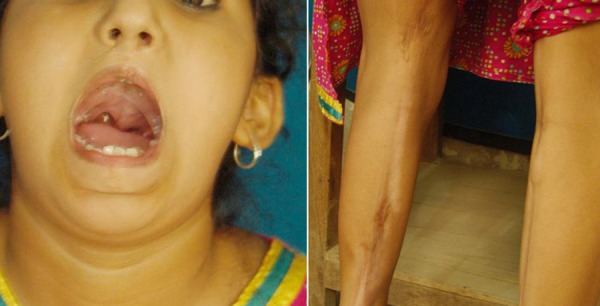
Figure 1: Index case showing repaired cleft lip, cleft palate and released pterygium.

Her father had the same congenital anomalies for which he had multiple surgeries (Fig. 2). Her younger sister also manifested popliteal pterygium syndrome anomalies (cleft lip, cleft palate, lower lip sinus and bilateral popliteal pterygium) (Fig. 3).

**Figure:**
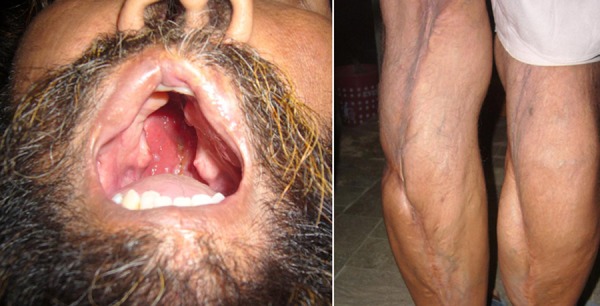
Figure 2: Father of the index case.

**Figure:**
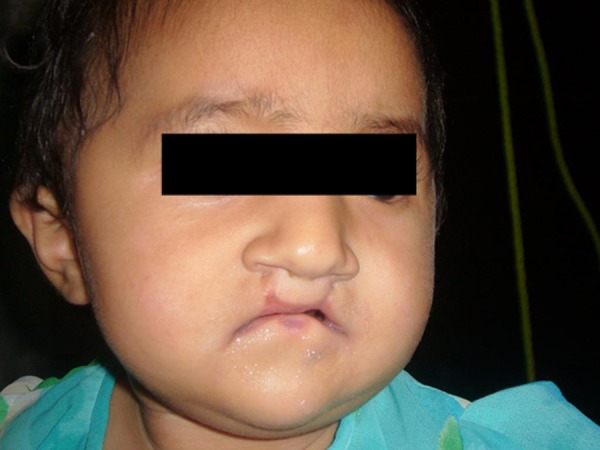
Figure 3: Younger sister of the index case.

The index girl achieved all developmental milestones normally. She has learned to walk and run but she cannot put her heals fully plantigrade. Pharyngoplasty was performed at last admission and patient discharged. She is on regular follow up.

## DISCUSSION

The popliteal pterygium syndrome is a congenital malformation that includes orofacial, cutaneous, musculoskeletal, and genital anomalies. The minimal diagnostic criteria for popliteal pterygium syndrome are any three of the following namely cleft lip/palate, popliteal pterygium, paramedian lower lip sinuses, genital and toe nail anomalies [5].

Cleft palate with or without cleft lip has been found to be the most frequent anomaly in popliteal pterygium syndrome, being present in 91 to 97% of cases. Cleft lip occurs in 58% and lower lip pits or sinuses occur in 45.6% of cases. Popliteal webbing in 58%, syndactyly in 50%, genitourinary anomalies in 37% and nail anomalies in 33% of cases. Other reported clinical features include syngnathia, ankyloblepharon, talipes, and digital reduction defects. There is no growth disturbance and intelligence is usually normal. All cases in our reported family had cleft lip, cleft palate, lower lip sinus and bilateral popliteal pterygium which fulfill the PPS diagnostic criteria.

The genetic locus for PPS has been localized to chromosome 1. The disorder is inherited in an autosomal dominant manner and is due to a mutation of the IRF6 gene. Mutation of RIPK4 gene on chromosome 21 has been identified to be the cause of autosomal recessive PPS. Most reported cases are sporadic; advanced parental age is found in a number of these cases, suggesting new mutations [6]. In our reported family, the father has PPS and mother is normal. The parents and siblings of the father are normal which suggests a new mutation in the father for PPS and then transmitted to his daughters in an autosomal dominant manner. Differential diagnosis includes two groups; the syndromes with similar orofacial anomalies [7] and disorders with similar limb defects [8]. The first group includes cleft lip and palate syndromes, van der Woude's syndrome, which presents with paramedian lower lip pits and cleft lip/palate and is inherited as an autosomal dominant trait [7,8]. The second group includes lethal PPS and PPS with ectodermal dysplasia. Both are autosomal recessive disorders. Although both conditions feature a cleft lip/palate, syngnathia, and popliteal pterygium, they are clinically distinguishable from the autosomal dominant case. Lethal PPS is differentiated by the presence of microcephaly, corneal aplasia, ectropion, bony fusions, hypoplastic nose and absent thumbs, while PPS with ectodermal dysplasia is differentiated by the presence of woolly hair, brittle nails, ectodermal anomalies, and a fissure of the sacral vertebrae [8].

Patients have to undergo a series of operations for correction of the congenital anomalies. In the newborn period the ankyloblepharon and oral synechia are corrected to enable eye opening and proper feeding. Cleft lip and palate repair are done in consecutive sessions starting around 2-3 months of age. Early surgical intervention for the popliteal webs appears to be important with respect to long term results. During the operation special attention needs to be given to the vessels and nerves within the pterygium. Postoperatively, plaster casts and physiotherapy are used to ensure good long term results.

The role of MRI in evaluating the normal or abnormal position of the popliteal artery and peroneal nerve provides useful information for preoperative planning for surgical correction of the popliteal pterygium. Operations include excision of the fibrous band, mobilization of nerves and vessels and Z-plasty of the skin [9]. Recurrence of flexion contracture is noted in some cases. Gradual soft tissue lengthening with an Ilizarov external fixator can be one of the optimal procedures when excision of a fibrous band and Z-plasty are not possible due to severe adhesion of the nerves and vessels into a fibrotic band [10].

## Footnotes

**Source of Support:** Nil

**Conflict of Interest:** None declared
